# Hot or Not: The Effects of Exogenous Testosterone on Female Attractiveness to Male Conspecifics in the Budgerigar

**DOI:** 10.1371/journal.pone.0074005

**Published:** 2013-08-12

**Authors:** Stefanie E. P. Lahaye, Marcel Eens, Veerle M. Darras, Rianne Pinxten

**Affiliations:** 1 University of Antwerp, Research Group Ethology, Antwerp, Belgium; 2 KU Leuven, Laboratory of Comparative Endocrinology, Leuven, Belgium; Arizona State University, United States of America

## Abstract

An increasing number of studies indicate that not only females but also males can be selective when choosing a mate. In species exhibiting male or mutual mate choice, females may benefit from being attractive. While male attractiveness is often positively influenced by higher plasma levels of the androgenic hormone testosterone, it has been shown that testosterone can masculinise female behavior and morphology in several bird species, potentially rendering them less attractive. In this study, we investigated whether female budgerigars, *Melopsittacus* *undulatus* , suffer from increased plasma testosterone levels through a negative effect on their attractiveness to males. We experimentally increased plasma testosterone levels in testosterone-treated females (T-females) compared to controls (C-females) and allowed males to choose between a T- and a C-female in a two-way choice situation. Although testosterone treatment significantly affected female behavioral and morphological characteristics, males did not show a significant difference in preference between T- and C-females. These results suggest that experimentally increasing testosterone levels in females does not appear to influence male preference during initial mate choice. Our findings indicate that selection for higher levels of testosterone in male budgerigars is probably not constrained by a correlated response to selection causing negative effects on female attractiveness during initial mate choice. Evaluating whether or not a potential constraint may arise from negative testosterone-induced effects on other fitness related traits in females requires further work.

## Introduction

While mating preferences and mate choice have been extensively studied in females, less attention has been paid to male mate choice. Most evidence for male mate choice comes from species in which females vary largely in fecundity, such as amphibians and fish, but it is becoming increasingly clear that male mate choice is not limited to these taxa [1]. Especially in socially monogamous species in which both sexes substantially invest in reproduction, males may benefit from being selective when choosing a partner [2–4]. In birds, monogamy is the most common mating system and males often provide paternal care [5]. Accordingly, evidence for male or mutual mate choice has been found in many bird species (e.g. [1,6–9]). Experimental studies have shown that males may express preferences based on traits that signal female fecundity or reproductive status (e.g. dietary condition in zebra finches, *Taeniopygia* *guttata* , brooding behavior in Japanese quail, *Coturnix* *japonica* [6,10]), as well as traits whose signaling content is less well understood (e.g. crest length in crested auklets, *Aethia* *cristatella* , breast patch size in rock sparrows, *Petronia* *petronia* [11,12]). As indicated by these studies, there is growing support for the assumption that not only females but also males can show preferences for attractive traits and selectively choose their partners based on these traits. In such a mating system, attractive females may form pair bonds more easily and may acquire mates of higher quality or mates which provide more paternal investment [8,13,14]. Hence, in the case of male mate choice, female attractiveness may be an important mediator of female reproductive success [8,15].

With respect to males, attractiveness to females seems to be based on a variety of traits, including song, courtship dances and color displays (e.g. [16–19]). The expression of these traits is often dependent on plasma levels of the androgenic steroid hormone testosterone (T), with males that show higher plasma T levels expressing more elaborate characteristics (e.g. [20–22]; but see [23]:). This suggests that males may benefit from higher plasma T [24–26] because it renders them more attractive to females [27]. However, selection for higher plasma T levels, even when beneficial to males, may be constrained if a correlated response to selection is harmful to females. Such an intralocus sexual conflict can arise when the genes that underlie trait expression are shared between the sexes [28,29]. Comparative studies in several taxa, including birds, have found that male and female androgen expression co-varies, indicating a strong intersexual genetic correlation with respect to plasma T levels and hence a potential intralocus sexual conflict [30–32]. Investigating the assumption that elevated T levels are indeed detrimental to females is the first step in evaluating whether sexually antagonistic selection potentially hinders evolution to higher plasma T levels in males.

Experimental studies have shown that elevated plasma T concentrations can cause changes in female morphology and behavior in several bird species (reviewed in 30). Exogenous T can affect female phenotype by inducing or increasing the production of male-typical behaviors, such as vocalizations (e.g. [33–35]) and other courtship behaviors [35–37], or by altering female color displays [34,38]. Such effects may be negative for females if they cause a decrease in their attractiveness to potential mates. For example, it has been shown that female zebra finches, *Taeniopygia* *guttata* , supplemented with T express more brightly colored beaks [39], while males normally prefer females with intermediately colored beaks [40]. This indicates that female zebra finches with higher T levels may be less attractive to males. It has also been found that T-treated female spotless starlings, *Sturnus* *unicolor* , produce a lower number of extra-pair offspring [41]. Although the underlying mechanisms are not yet clear, one possible explanation for these results may be that T-females are less successful with respect to extra-pair copulations, possibly through an effect on female attractiveness ( [41]; but see [42]:). Moreover, male dark-eyed juncos, *Junco* *hyemalis* , seem to associate more with controls compared to T-treated females (Parker-Renga et al., unpublished results in [30]), and T-treated female leopard geckos, *Eublepharis* *macularius* , experience more intersexual aggression [43], indicating that T-treated females are less attractive to males in both species. Although these studies suggest that elevated plasma T levels potentially affect female attractiveness, there is little direct evidence of such an effect.

The budgerigar, *Melopsittacus* *undulatus* , is a small monogamous parrot species which forms long-lasting pair-bonds [44]. In this species, males show T levels which are four to ten times higher than female T levels [35,45]. The expression of several male behavioral and morphological traits that are important in determining female mate choice and breeding success in the budgerigar (e.g. courtship behaviors [46,47]: and color displays [45,48]:), is enhanced by treating males with exogenous T [45,49,50]. This indicates that male budgerigars with higher plasma T levels may experience an increase in reproductive success through a positive effect on traits such as mate acquisition behavior, courtship effort and attractiveness to females. Male budgerigars show preferences based on the color of the cere, a fleshy structure over the beak which is brown in females and blue in males. Paired males prefer extra-pair females with dark brown ceres over females with a lighter cere color and males paired with females with darker brown ceres show more courtship behavior towards their partner [51,52]. Interestingly, it has been shown in two independent studies that the behavior and morphology of female budgerigars supplemented with T change to the male-typical phenotype [35,53], thereby potentially rendering them less attractive.

In this study, we investigated experimentally whether exogenous T affects female attractiveness during initial mate choice in the budgerigar. We increased plasma T levels to the male-like level in experimental females (T-females) compared to controls (C-females) by supplementing them with a subcutaneous T-implant. Then we tested the preferences of unpaired male budgerigars for T-females compared to C-females in a two-way choice situation. If exogenous T decreases female attractiveness during initial mate choice, we would expect males to show a preference for C-females over T-females.

## Material and Methods

### Ethics statement

The budgerigars used in this study were domesticated animals which were used to human presence. Because budgerigars are social birds, we always allowed the birds at least vocal interactions. We bled the birds to obtain approximately 300 µL blood from the alar vain, which represents less than 1% of the body weight and does not cause adverse effects [54]. The females behaved normally within a few minutes after blood sampling. We used Xylocaine (10% spray) to locally anaesthetize the females during the implantation procedure. Implantation did not cause more apparent distress than blood sampling. We found no immediate adverse effects after treatment or later on. Implantation (see ‘implantation procedure and blood sampling’) did not affect female body weight or survival and previous experiences have shown that T- and C-females reproduce successfully in subsequent breeding seasons (unpublished data). In addition, we did not observe abnormal behavior performed by the T- and C-females during the daily routine checks or during the preference tests. Handling time was minimized and did not exceed 3 min per individual for all procedures. All experimental procedures were performed in agreement with the Belgian and Flemish laws and were approved by the ethical committee of the University of Antwerp (ID number 2011/25).

### Study species and housing

We randomly selected 32 female and 32 male budgerigars with a green plumage. The birds had been obtained from local breeders as juveniles and had been maintained in our captive stock for two years. All birds were of similar age (two years old) and had previous breeding experience. Before the onset of the experiment, they were housed in two single-sex outdoor aviaries (8m wide x 2.5m deep x 2.3m high) for at least nine months. Two weeks before implantation (see ‘implantation procedure and blood sampling’), the females were housed individually in indoor cages (60cm wide x 40cm deep x 50cm high) in one single-sex room with auditory but no visual contact. All females were in breeding condition and expressed normal cere color of adult females in breeding condition. Four weeks before the start of the preference tests, the males were moved to similar indoor cages in a single-sex room. When housed indoors, the birds were maintained on a light regime of 15:9 (L: D). Food (commercial budgerigar seed mix, Van Camp, Belgium) and water were provided ad libitum.

### Implantation procedure and blood sampling

On 23 June 2011, females from both treatments received a silastic tubing implant (Degania silicone; length: 7 mm, 1.47 mm i.d., 1.96 mm o.d.). Implants were packed with 4 mm of crystalline T (Fluka 86500, 3.52 ± 0.03 mg) or left empty, and sealed on both ends with silastic glue. Before treatment, females were matched for blue chroma of the cere (see ‘female traits’) and assigned to a female stimulus set that consisted of two matched females. Assignment to either the testosterone group (T-females, n=16) or the control group (C-females, n=16) within a female stimulus set was done ad random. Implants were inserted subcutaneously along the left flank and the incision was sealed with tissue adhesive (Histoacryl, B. Braun). Implants of this size were selected based on previous work in female budgerigars, because they successfully increase plasma T to the male-like level within two weeks after implantation [35]. Blood samples were collected immediately before implantation and seven weeks after implantation, immediately following the preference tests. Sampling immediately following the tests allowed us to obtain a reliable measure of plasma T levels during the preference test, while preventing possible disturbance during the testing due to handing and blood sampling. Blood samples were centrifuged at 7000 rpm for 10 min within two hours of sampling. The plasma fraction of 50-150 µL was collected and stored at -70° C until a hormone assay was performed. Plasma T concentrations were quantified by radioimmunoassay (RIA) using a commercial double antibody system purchased from MP Biomedicals (Solon, Ohio). Our hormone assay techniques have been reported previously [55]. Briefly, 500 µL of a 50/50 mixture of cyclohexane/ethyl acetate was added to 50 µL plasma. After incubation, the tubes were extracted twice and the organic phase was transferred to a new tube and dried by vacuum centrifugation. The dried samples were dissolved in 25 µL steroid diluent buffer and further treated following the protocol of the RIA kit. The specification sheet provided by the company indicates that the primary antibody used in this assay does not cross-react significantly with other androgens beside T (5-dihydrotestosterone: 3.4%; 5-androstane-3,17-diol: 2.2%; 11-oxo-testosterone: 2%; all other steroids: <1%). T standards ranged from 0.1 ng/mL to 10 ng/mL but the effective detection limit could be extended to 0.05 ng/mL owing to the concentration effect of the extraction procedure. The intra-assay coefficient of variation was 4.6-9.1% (medium - low/high concentrations) and all samples were measured within the same assay.

### Female traits

We measured four female characteristics (body weight, interest score, UV chroma and blue chroma of the cere) that may potentially determine male preference in budgerigars. Body weight was measured before implantation and immediately before the preference tests. Body weight may predict female reproductive status or fecundity [56,57] and therefore males may show preferences to female body weight [1,58].

In species in which mate attraction coincides with the performance of certain behaviors, preference measured during choice tests may be affected by the behavior of the stimulus animals [27,59]. It has been shown that treating female budgerigars with T causes them to perform high levels of male-typical (e.g. socio-sexual behavior and vocalizations) but not aggressive behaviors [35,53]. However, in this study, only one T-female performed socio-sexual behavior twice during the preference tests and no females were observed performing aggressive behaviors. During preliminary analyses we noticed that some females regularly faced the male during the preference tests, while others seemed to largely ignore the male. As budgerigars are a highly social species, preference may be influenced by such association behavior or interest. We therefore scored the position of the females (on the perch in front of the male while facing the male versus in another position) each time the male visited them during the preference tests (see ‘preference tests’). The scoring of the females was done at the same time intervals as the scoring of the males (see ‘preference tests’). We calculated the female’s interest score during a preference test as the proportion of times the female was facing the male when he visited her. Only preference tests in which the male visited both females at least ten times over the two observation periods were used. All preference tests in this study met this criterion.

In the budgerigar, cere color may be an important cue in male mate choice. Females with darker colors receive more courtship behavior from their partner and such females are preferred as extra-pair partners over females with light cere colors [51,52]. We therefore estimated the effect of cere color on male preference using values for UV chroma and blue chroma of the cere which we calculated from reflectance spectrophotometry data for all females. We included UV chroma of the cere as it has been shown that UV reflectance may be important in determining female preference and signaling male quality in the budgerigar [60,61]. One day before implantation and one week before the preference tests, the color of the cere was measured with an USB4000 spectrophotometer (Ocean Optics, Duiven, The Netherlands), using an Ocean Optics DH-2000 BAL deuterium/halogen lamp. Before the measurement session we took a dark current measurement on the cere of a randomly selected live bird and a white standard reference measurement (WS-1, Diffuse Reflectance Standard, Ocean Optics, Duiven, The Netherlands) for calibration purposes [62]. Next, the left side of the cere of all individuals was measured three times by the same person. From the measurements, we calculated UV chroma and blue chroma as the proportion of total reflectance occurring between respectively 320-400nm (R_320-400_/R_320-700_) and 400-500nm (R_400-500_/R_320-700_) [63]. For each individual, we first calculated the UV chroma and blue chroma separately for the three spectra that were measured and then we calculated the means of these three values which were used in the statistical analyses.

### Preference tests

**Figure 1:**
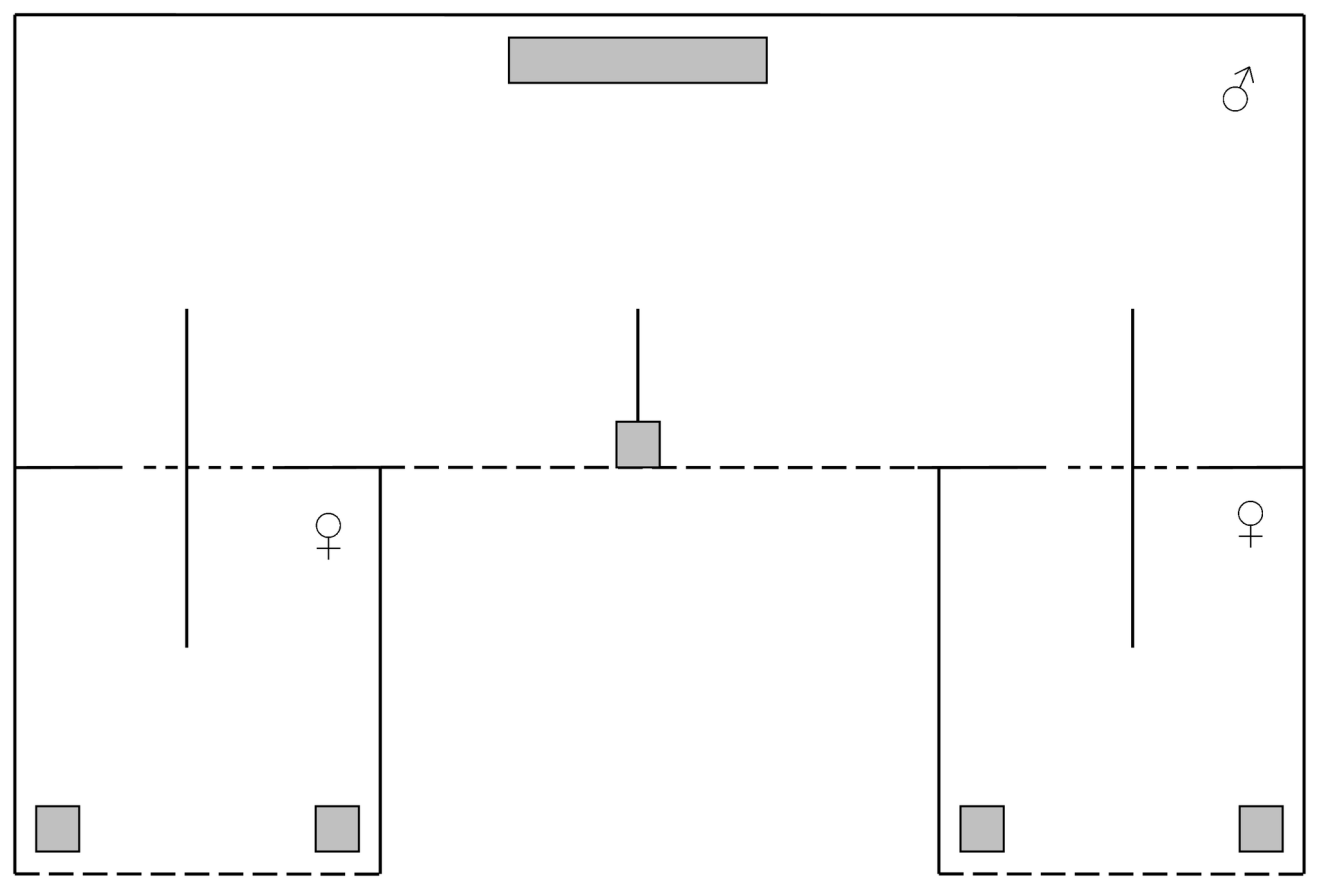
Diagram of the choice test apparatus used in the experiment. The dark gray lines represent the perches. The light gray squares represent the food and water containers. Dotted lines represent grated cage walls.

Male preference was tested in a two-way choice test during four consecutive days (05 -08 August 2011). We allowed the males to choose between a T-female and a C-female of a female stimulus set. Eight choice tests were conducted simultaneously in two separate rooms between 0900 and 1300 hours local time. The choice test apparatus consisted of two small cages (35cm wide x 35cm deep x 50 cm high) that were placed frontally on either side of a large central cage (120cm wide x 40cm deep x 50cm high; Figure 1). The females had access to one perch. The male had access to one perch on the side of each female and one centrally positioned perch. The females were visually separated and the male could only see the females when he was positioned in the choice area (directly in front of the females’ cages), preventing him from seeing the females simultaneously and from seeing the females while positioned in the no-choice area [61]. All individual set-ups were visually separated from each other. The experimental cages were illuminated by artificial full spectrum light (Philips True Light, 58W/5500) and natural light through windows. Food and water were provided ad libitum at all times. A similar set-up has been used in previous studies investigating mating preferences in different species, including budgerigars (e.g. [47,61,64]).

The afternoon of the day preceding the preference tests, the T- and C-female of a stimulus set were moved to the small cages, each small cage containing one female, and a male was placed in the large central cage. The birds were allowed to adjust to the new environment overnight. During this habituation period, the birds were visually but not acoustically separated. Because budgerigars are a highly social species, we played back budgerigar calls during the first hour of the habituation period to minimize stress and to better habituate the birds [61,65]. The following morning, the artificial lights were turned on, the partitions that visually separated the female from the males were removed and the birds were allowed a habituation period [66]. After 45 min, the position of the females was switched and a video-recording of 45 min was made to test male preference. During preliminary testing of our experimental set-up, we noticed that in many cases the test birds initially showed little mobility and only interacted with one stimulus bird without visiting the second. By switching the position of the females before the first recording, we could increase the probability that all males had interacted with both females at the start of the actual test phase. A second 45-min recording was made after switching the position of the females a second time to control for side preferences of the male.

We analyzed 30 min of each recording, excluding the first 5 min and last 10 min to avoid potential disturbance caused by manually switching the test cages, by entering and leaving the testing facility and by starting and stopping the cameras. We estimated preference by scoring the position of the male every 10 seconds (left choice area, right choice area or central), yielding a total of maximum 180 association score points per observation period for both females. We calculated the total association score for a female by summing the scores obtained for the female over the two observation periods of a preference test. This score was used in further data analysis. We considered a preference test to be successful if the male was responsive during the preference test (i.e. the male spent at least 50% of the choice period (≥180 association score points) in the left or right choice area [27,59]). A male was considered to exhibit a preference when the association score for both females differed significantly (one-tailed binomial test, *P*<0.05). Each of the 16 female stimulus sets was presented during two different preference tests to test the preference of 32 males. The preference test of one male was not successful and this preference test was discarded. Finally, testing resulted in 31 successful preference tests. Males were always tested with unrelated females that they were not familiar with and we made sure that all females that were present in the same test room during a preference test were unfamiliar with all males.

### Data analysis

We analyzed all data using the statistical package SAS^®^ 9.2 (SAS Institute, Cary, NC, 2008). Data were checked for normality and, if not normally distributed, log (x + 1) transformed. All proportions were arcsin-square-root transformed. To investigate the effects of treatment (T or C) on the mean interest score of the females based on the two preference tests after implantation, a student’s t-test was used. We used repeated measures ANOVA to investigate the effects of treatment on plasma T levels and on female traits (body weight, UV chroma and blue chroma of the cere). Treatment, sampling (before and after implantation) and their interaction were included as fixed effects. Female identity was included in the repeated statement. Because repeated observations were made on single individuals, residual values may be correlated. Therefore, we tested several covariance structures (i.e. compound symmetry, serial autocorrelation and unstructured) to select the best fitting regression model based on BIC values. The Satterthwaite correction was used to adjust the degrees of freedom [67]. Within all SAS models these analyses were followed by post-hoc comparisons using t-statistics with adjusted *P*-values (*P*_*a*_) for multiple testing using Tukey corrections. We always started with the complete model and subsequently dropped step by step all non-significant terms. At each step, the term that gave the smallest contribution (largest *P* value) was excluded.

Male preference in relation to treatment was investigated using two different tests. First, we tested whether there was a difference in the number of males preferring the C-female versus the T-female using a one-tailed binomial test. Then, we calculated the proportion of association scores for the C-females as the association score of the C-female over the total number of score points of active choice (=sum of association score points for the T- and C-female). These proportions were compared to proportions of 0.5 as expected under random conditions [64]. By including the proportion of association scores instead of the absolute scores, the fact that the scores of the T- and C-female of a stimulus set are not independent is taken into account. Treatment, test (first or second preference test) and their interaction were included as categorical fixed effects. Female identity was included in the repeated statement to control for the fact that each female was used to test the preference of two different males. To evaluate whether the T- and C-females of a stimulus set differed for body weight, interest score, UV chroma and blue chroma of the cere, we used paired t-tests. We also tested whether differences in male preference were correlated with female traits (body weight, UV chroma and blue chroma of the cere and interest score during the according preference test) using a repeated measures ANOVA. The proportions of association score points for the C-females were included in the model as dependent variables. For each trait we calculated the difference between the C- and T-female of a stimulus set by subtracting the value of the T-female from the value of the C-female. These differences were included in the model as covariates and female stimulus set was included in the repeated statement. Values are reported as mean ± SE. Significance was calculated at the *P*<0.05 significance level.

## Results

### Plasma T levels

Implantation significantly increased plasma T concentration in T-females but did not significantly affect T concentrations of C-females (treatment x sampling interaction: F_1,26.7_=20.01, *P*<0.0001). Before manipulation, plasma T concentration did not differ between treatments (*t*_44.9_=0.23, *P*_*a*_>0.99, table 1) while after implantation, T-females had significantly higher plasma T levels than C-females (*t*_45_=-5.77, *P*_*a*_<0.001, table 1).

**Table 1:** Comparison of plasma T levels, body weight, UV chroma of the cere, blue chroma of the cere and interest score (mean ± SE) between T- and C-females before and after treatment.

		T-female	C-female
plasma T (ng/mL)	before	0.27 ± 0.10 ^a^	0.33 ± 0.17 ^a^
	after	2.05 ± 0.30 ^b^	0.11 ± 0.02 ^a^
body weight (g)	before	41.69 ± 1.29^c^	41.06 ± 1.58 ^c^
	after	47.06 ± 1.80 ^d^	43.31 ± 1.91 ^c,d^
UV chroma of the cere	before	0.14 ± 0.006^e^	0.14 ± 0.006^e^
	after	0.12 ± 0.01^e^	0.14 ± 0.006^e^
blue chroma of the cere	before	0.22 ± 0.005 ^f^	0.22 ± 0.006 ^f^
	after	0.26 ± 0.01 ^g^	0.22 ± 0.008 ^f^
interest score	before	*NA*	*NA*
	after	39.68 ± 3.34 ^h^	27.08 ± 2.92 ^i^

Values not connected by the same letter are significantly different.

### Female traits

Treatment significantly affected body weight (treatment x sampling interaction: *F*_1,30_=5.13, *P*=0.031, table 1). T- and C-females did not differ significantly before or after treatment (before: *t*_35.8_=-0.27, *P*_*a*_=0.99; after: *t*_35.8_=-1.62, *P*_*a*_=0.38). Females of both treatments experienced an increase in body weight but this increase was only significant for T-females (C-females: *t*_30_=2.31, *P*_*a*_=0.12; T-females: *t*_30_=5.51, *P*_*a*_<0.0001). Treatment also significantly affected cere color (treatment x sampling interaction: *F*_1,30_=5.91, *P*=0.021, table 1). T-females showed a significant increase in blue chroma of the cere (*t*_30_=3.61, *P*_*a*_=0.0058) while in C-females blue chroma did not differ before and after treatment (*t*_30_=0.17, *P*_*a*_>0.99). Only after treatment, T- and C-females differed significantly for blue chroma of the cere (before: *t*_58.8_=-0.59, *P*_*a*_=0.93; after: *t*_58.8_=-3.78, *P*_*a*_=0.0037). UV chroma of the cere was not affected by T-treatment (treatment: *F*_1,30_=1.03, *P*=0.32; sampling: *F*_1,31_=3.79, *P*=0.061; treatment x sampling interaction: *F*_1,30_=1.35, *P*=0.25, table 1). T-females had a significantly higher mean interest score than C-females (*t*_30_=-2.82, *P*=0.008, table 1).

### Preference tests

**Figure 2:**
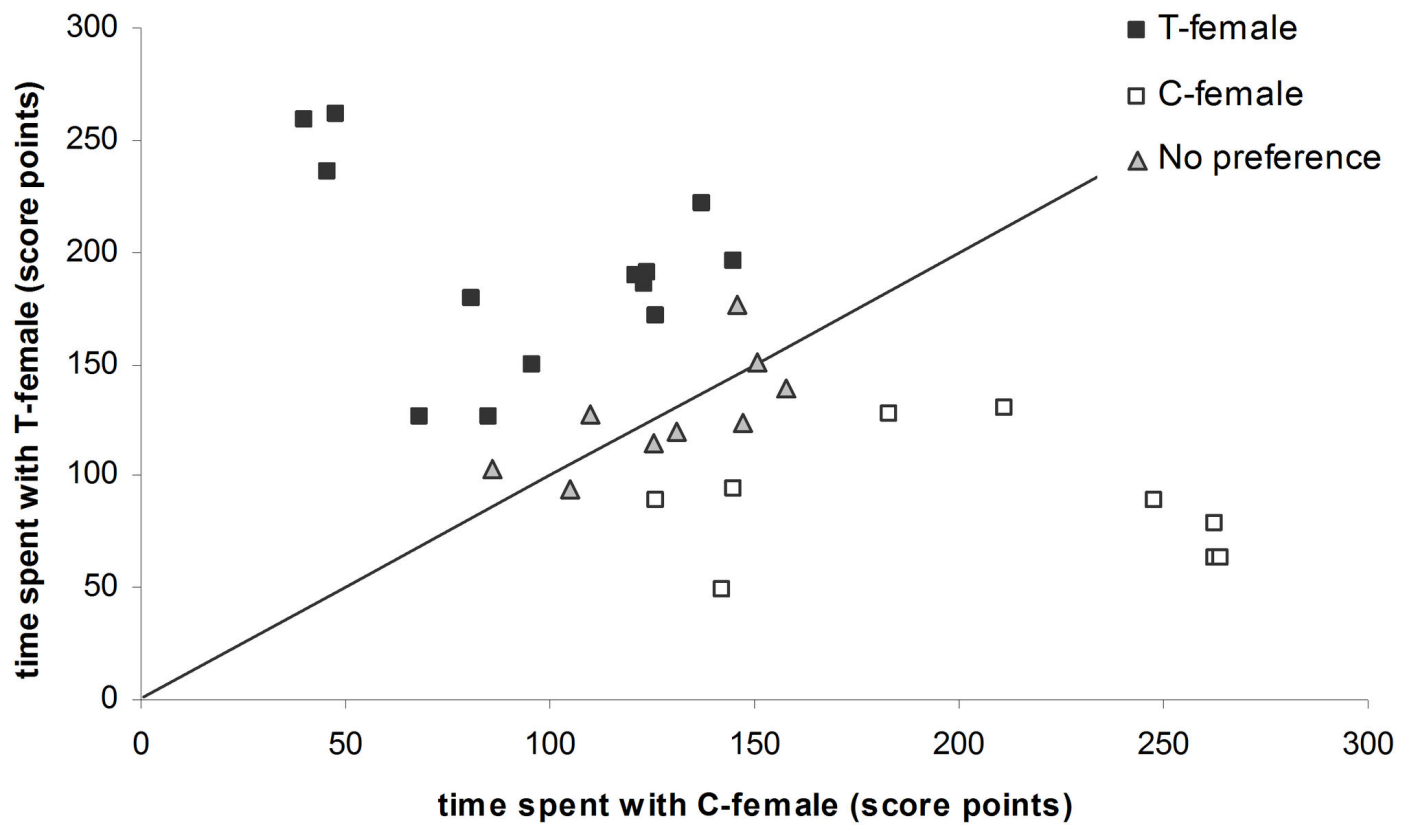
Male association patterns based on association score points according to female treatment group. Association score points above and under the 45° line represent respectively males that showed a significant preference for the C-female (open squares, n=9) and for the T-female (black squares, n=13) while association score points near the 45° line represent males that did not show a significant preference (grey triangles, n=9).

**Figure 3:**
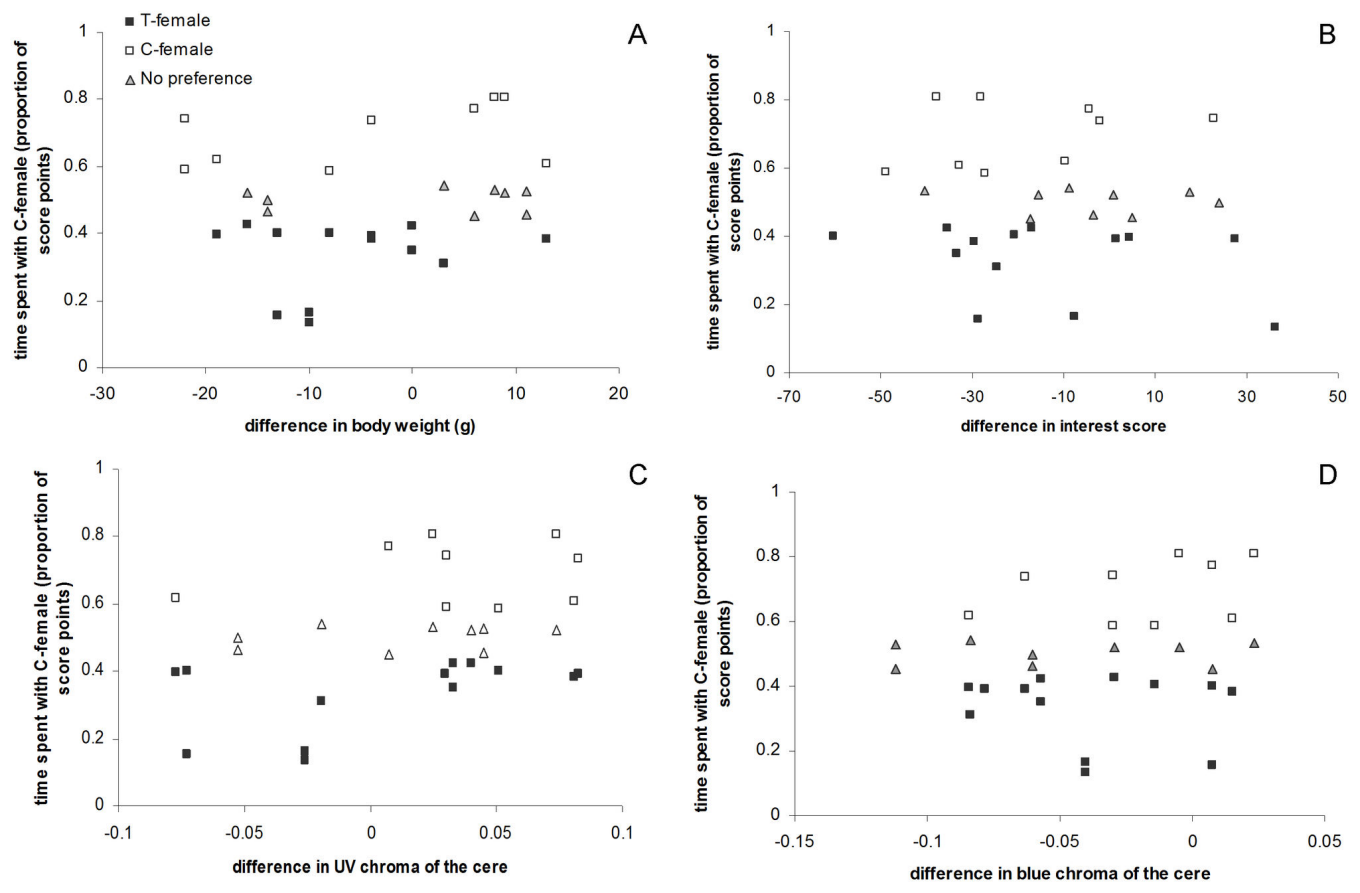
Male association patterns based on the proportion of association score points for C-females in relation to the difference between the C- and T-female of a stimulus set for four female traits: A) body weight, B) interest score during the according preference test, C) UV chroma of the cere and D) blue chroma of the cere. As all female stimulus sets were used to test the preference of two different males, each set is represented twice (two successful tests, n=15) or once (one successful test, n=1) in the graphs. Negative values indicate that the T-female of the stimulus set expressed a higher value for the trait than the C-female. Open squares indicate that the C-female (n=9) and black squares that the T-female (n=13) was preferred and gray triangles indicate that there was no significant preference (n=9).

Out of 31 successful preference tests, nine times the males showed no significant preference, nine times the C-female was preferred and 13 times the T-female was preferred (Figure 2). The proportion of males preferring the T- or C-female did not differ significantly (χ^2^_1_ = 0.73, *P*=0.39). The proportion of association scores for the C-females did not differ significantly from a proportion of 0.5 expected under random conditions (F_1, 30.4_=0.15, *P*=0.70). The effect of preference test was not significant (F_1, 30.8_=0.85, *P*=0.36) and neither was the treatment* test interaction effect (F_1, 29.9_=0.84, *P*=0.37). Paired t-tests revealed that, irrespective of treatment, the females of the stimulus sets differed significantly for all four female traits (body weight: *t*_15_=5.56, *P*<0.0001, mean interest score: *t*_15_=4.96, *P*=0.0002; UV chroma of the cere: *t*_15_=4.62, *P*=0.0003; blue chroma of the cere: *t*_15_=-5.35, *P*<0.0001). For interest score and blue chroma of the cere, the T-females of the stimulus sets expressed significantly higher values (interest score: *t*_30_=-2.84, *P*=0.008; blue chroma of the cere: *t*_15_=-3.66, *P*=0.0023), while the differences between the females of the stimulus sets for UV chroma of the cere and body weight were not significantly predicted by treatment (UV chroma of the cere: *t*_15_=1.22, *P*=0.24; body weight *t*_15_=-1.33, *P*=0.20). The proportions of association scores for the C-females was not affected significantly by the difference between the T-and C-females of the stimulus sets in body weight (F_1, 11.7_<0.01, *P*=0.96), interest score (F_1, 24.9_=0.04, *P*=0.84) or blue chroma of the cere (F_1, 13.3_=0.30, *P*=0.60), but there was a trend that males preferred females with higher values for UV chroma of the cere (F_1, 13.8_=3.81, *P*=0.071, Figure 3).

## Discussion

In this study, we successfully increased plasma T concentrations in T-females to the male-like level, thereby influencing morphological and behavioral characteristics, and we tested male preference for T-females compared to C-females in a two-way choice test.

### Elevated T and male preference

The number of males preferring T- and C-females did not differ significantly. There was also no significant difference between the association scores of the males for the T- and C-females, irrespective of the statistical approach. Our data indicate that female plasma T levels did not influence male preference, although they were experimentally increased to the male-like level. Hence, our data suggest that in the budgerigar, female attractiveness to males during initial mate choice is apparently not greatly influenced by exogenous T.

The result of our study is remarkable because a vast body of literature indicates that T can have masculinising effects on the behavior and the morphology of females in several species [30], including in the budgerigar [35,53], which may potentially render them less attractive to males. Possibly, the males from our study did not show a preference for females of one treatment over the other because they were unable to discriminate between T- and C-females. The females of a stimulus set did not differ with respect to age and UV chroma of the cere was not affected by T treatment but T- and C-females did differ in behavioral and morphological characteristics. T-females showed significantly higher interest scores and they expressed significantly higher values for blue chroma of the cere than C-females, which makes it unlikely that the males could not discriminate between the females of both treatment groups. It is also possible that there are limitations to how accurately male preference can be inferred from our experimental set-up [68,69]. However, preferences measured in the same species, using similar methods, have been shown to be repeatable between studies [61,64], which indicates that this method is useful to measure reliable preferences in the budgerigar. Given the fact that the birds could not physically interact in our experimental set-up, there is also a possibility that we measured social preference rather than sexual preference [70]. We are convinced that this is unlikely as previous two-way choice tests indicate that budgerigars show repeatable preferences for birds of the opposite sex that express specific traits rather than simply sitting close to a random conspecific [61,64]. In addition, for budgerigars it has been found that female preference, also measured in a design similar to the one we used, is reflected in pairing patterns [47,71]. These findings support the notion that our measure of sexual preference is likely to be reliable. Moreover, in the budgerigar, pairing usually takes place within hours and after initial pair bond formation both members of the pair remain in close proximity of each other [44]. This further indicates that association scores are likely to reflect sexual preferences in this species [6].

### Female traits and male preference

We found no effect of difference between the T- and C-female of the stimulus sets for interest score, body weight, UV chroma or blue chroma of the cere on the association scores of the females. Female budgerigars have been shown to express mating preferences based on male behavioral traits, namely warbling song (e.g. [47]). As far as we are aware, it has not been investigated whether female behavioral traits are important for determining male preference in budgerigars. Our results suggest that this is not the case for interest score. It may be that other behavioral traits such as vocalization similarity between the members of a potential future pair [71] are more important in determining male preference.

T-treated females expressed significantly bluer ceres than controls. Although female cere color was not fully masculinized, blue chroma of the cere of T-females but not of C-females was on average more similar to the male than the female condition (unpublished data). In the budgerigar, cere color may signal female reproductive status as it has been suggested that females with darker brown ceres circulate higher levels of estrogens and have more developed ovaries ( [52,72]; but see [73]:). Hence, it is possible that males benefit from choosing females with darker brown ceres because they are more fertile. In accordance, a previous study found that males having to choose between extra-pair females that had their cere colors experimentally manipulated using light blue or dark brown non-toxic paint, prefer to associate with the latter [51]. Our findings contrast with this study. If male budgerigars show preferences based on multiple cues [74], it could be that cere color is less important in determining male preference than other cues. These cues may not have been affected or even enhanced by T treatment, thereby masking a preference for cere color in our study. It may be that these other cues, for example the performance of certain intrasexual behaviors, could not be used by the males in the previous study due to the experimental set-up as the females were presented behind one-way glass [51]. Alternatively, it is possible that the difference in preference for female cere color was caused by differences in the social contexts of the males. We tested the preference of unpaired males while in the previous study the preference of paired males for potential extra-pair females was tested [51]. Colorations of bare parts (e.g. ceres, bills, legs) are dynamic traits which can provide current information on an individual’s condition or state [18,75,76]. Therefore, it may be that such colors are used to estimate the condition or state of an individual at any point in time rather than being specifically used as a cue during initial mate choice [18]. While it has been found that females may use bare-part coloration of their mate as a cue to continuously adjust reproductive investment after paring (e.g. [77–79]), the function of female bare-part color on male reproductive decisions has been largely neglected. Research has shown that male blue-footed boobies, *Sula* *nebouxii* , use female dynamic foot coloration as a cue to modify parental effort and to adjust their frequency of intra-pair courtship and extra-pair behavior [80,81]. Paired male budgerigars have limited opportunity for pursuing extra-pair copulations [51]. Thus, if cere color indeed signals reproductive status, males would benefit from courting extra-pair females with dark brown cere color because they are more likely to be fertile. As in the budgerigar pair-bond formation is not necessarily associated with breeding [73], male preference at the time of pair-bonding may depend less on cues that signal reproductive status. The fact that males perform more courtship behavior towards females with dark brown ceres after pairing but not immediately after pair formation [52] may also be attributed to a dynamic signalling function of cere color. Our findings stress the need for increasing our understanding of the signalling functions of dynamic female ornaments and the effects of such ornaments on males.

We found a trend that males preferred to associate with females that expressed higher values for UV chroma of the cere, irrespective of T treatment. In male budgerigars, UV reflection is an honest signal of quality [60]. To our knowledge, it has not yet been investigated whether UV colors serve a similar signalling function in females. While it is widely accepted that female preference generates directional sexual selection on ornamental traits that signal male quality [82,83], it remains less clear whether or not ornamental traits can function to signal female quality. It is thought that the evolution of female ornamentation through male preference may be counterbalanced by a trade-off between the expression of costly ornaments and female fecundity [84]. However, in various bird species it has been found that males show preferences for more ornamented females (e.g. [12,85]). Furthermore, several studies indicate that female ornamental traits correlate with a variety of indicators of female quality and reproductive potential (e.g. female survival, competitive ability, offspring provisioning, clutch size, offspring quality and fledgling success [86–90];; see 91 for a review). These findings support the assumption that ornamental traits may have the potential to function as signals of individual quality, not only in males but also in females. Although in this study there was only a correlative trend that males preferred females based on ornamental UV coloration, our results suggests that also in budgerigars, female ornamental colors may signal important information during mate choice.

### Evolutionary implications

In males, higher T levels are generally considered beneficial, as they often correlate with male attractiveness, male mating success and number of extra-pair offspring [24–27]. Nevertheless, selection for higher T levels in males may be constrained if an intersexual genetic correlation gives rise to strong correlated selection pressures on females, with associated fitness consequences (e.g. [30,31]). In the budgerigar, males may benefit from higher plasma T levels as the expression of male behavioral and morphological traits that are related to mate acquisition, mating and attractiveness to females is enhanced by T treatment [45,49,50]. We found no effect of increased plasma T levels in females on male initial preference. This suggests that an evolutionary constraint on higher T levels in males through T-induced negative effects on female attractiveness seems to be of little importance in the budgerigar. However, it is possible that such negative effects may only become apparent in the later stages of pair formation. In addition, elevated T levels may also affect other fitness related traits in females, besides attractiveness during initial mate choice. For example, in some bird species, increasing T to the male-like level or within the high female physiological range has been found to interfere with female immunocompetence [34,38,92], egg laying [93–96] and maternal investment [94,95,97,98]. It remains to be investigated whether female budgerigars suffer similar costs from exogenous T, thereby promoting antagonistic selection with respect to plasma T levels. Along with identifying the traits that are sensitive to exogenous T, it is also necessary to increase our understanding of the pathways that are involved in T-induced effects on females. Until these pathways have been revealed, it cannot be ruled out that potential constraints on evolution are not caused by T itself but by other factors such as by-products of T metabolism [39].

In conclusion, we found that although T- and C-females differed significantly in some morphological and behavioral traits, male preference was not affected by T treatment. Female attractiveness to males during initial mate choice does not appear to be influenced by exogenous T in budgerigars. This indicates that selection for higher T levels in male budgerigars is probably not constrained by a correlated response to selection causing negative effects on female attractiveness during initial mate choice but it remains to be investigated whether elevated T levels are harmful to females by affecting other fitness related traits. 
